# Inhibition of Aberrant IGF-I Signaling in Diabetic Male Rat Retina Prevents and Reverses Changes of Diabetic Retinopathy

**DOI:** 10.1155/2019/6456032

**Published:** 2019-03-27

**Authors:** Gang Xi, Christine Wai, David Clemmons

**Affiliations:** Division of Endocrinology, Department of Medicine, University of North Carolina School of Medicine, Chapel Hill NC 27599, USA

## Abstract

Hyperglycemia results in inhibition of cleavage of integrin-associated protein (IAP) thereby allowing it to bind to SHPS-1 which results in pathophysiologic changes in endothelial function. This study determined if an anti-rat IAP antibody directed against the SHPS-1 binding site which disrupts IAP/SHPS-1 association could inhibit these pathophysiologic changes. The anti-IAP antibody inhibited IGF-I-stimulated SHPS-1, p52Shc, MAP kinase phosphorylation, and proliferation in endothelial cells. To determine if it could reverse established pathophysiologic changes in vivo, this antibody or normal rat IgG F(ab)2 was injected intraperitoneally for 6 weeks into rats that had diabetes for 4 weeks. Optical coherence tomography (OCT) showed that retinal thickness increased at 4 weeks and this increase was maintained in rats treated with the control antibody for an additional 6 weeks. The increase was reversed by anti-IAP antibody treatment (84.6 ± 2.0 compared to 92.3 ± 2.5 *μ*m, *p* < 0.01). This value was similar to nondiabetic animals (82.2 ± 1.6 *μ*m, *p*, NS). The anti-IAP antibody also decreased retinal vascular permeability (0.62 ± 0.12 vs. 0.96 ± 0.25%/g/h, *p* < 0.001). To determine if it was effective after local injection, this antibody or control was administered via intravitreal injection. After 3 weeks, retinal thickness increased to 6.4 ± 2.8% in diabetic rats, and IAP antibody treatment prevented this increase (0.8 ± 2.5%, *p* < 0.01). It also prevented the increase of retinal vascular permeability (0.92 ± 0.62 vs. 1.63 ± 0.99%/g/h, *p* < 0.001). Biochemical analyses of retinal extracts showed that the anti-IAP antibody inhibited IAP/SHPS-1 association and SHPS-1 phosphorylation. This resulted in inhibition of AKT activation and VEGF synthesis in the retina: changes associated with increased vascular permeability. We conclude the anti-rat IAP antibody disrupts IAP/SHPS-1 association and attenuates aberrant IGF-I signaling thereby preventing or reversing the progression of retinal pathophysiological changes.

## 1. Introduction

Diabetic retinopathy (DR) remains a major cause of severe vision impairment. Although the incidence is declining, the prevalence is increasing because of an aging population and the increase in the number of patients with diabetes [1]. Aging, duration of diabetes, and severity of hyperglycemia are the major driving variables [2]. Patients with hemoglobin A1c values > 8.0 have a significant long-term risk [3]. Analysis of the mechanisms involved and the potential approaches to medical therapy for diabetic retinopathy have utilized animal models of this condition. Rodent models have the advantages of being inexpensive, and they develop significant pathophysiologic changes over a relatively short time course [4]. However, a major disadvantage is that they do not develop neovascularization, a hallmark of late-stage human disease [5]. Nevertheless, compounds such as vascular endothelial cell growth factor (VEGF) antagonists which have been shown to inhibit pathophysiologic changes in rodent models also inhibit neovascularization in humans; therefore, rodent models continue to be utilized to study the early changes that occur in DR [6].

Growth factors other than VEGF have been implicated in retinopathy development. One factor that has been analyzed extensively is insulin-like growth factor-I (IGF-I) [7]. Retinal endothelial cells express IGF-I and IGF-I receptors [8], and in a mouse model of oxygen-induced retinopathy, IGF-I antagonists suppressed retinal neovascularization [9]. An endothelial cell specific knockout of the IGF-I and insulin receptors in mice protected against retinal neovascularization [10]. Transgenic overexpression of IGF-I in the retina resulted in several changes in mice that mimic human diabetic retinopathy including the development of acellular capillaries, pericyte dropout, and increased vascular permeability [11]. IGF-I induces VEGF in multiple in vitro and in vivo models, and studies have shown that the IGF-I-induced increase in VEGF activates the VEGFR2 receptor [9, 11–13].

Based on those studies, our laboratory has extensively analyzed IGF-I signaling in both vascular endothelial and smooth muscle cells maintained under hyperglycemic conditions. We have determined that high glucose downregulates the principal signal transduction element utilized by the IGF-I receptor: IRS-1 [14]. This leads to a signaling switch wherein the transmembrane protein, Src homology 2 (SH2) domain-containing protein tyrosine phosphatase substrate 1 (SHPS-1), is tyrosine phosphorylated in response to IGF-I, and this leads to aberrant activation of the MAP kinase pathway [15]. During hyperglycemia, the IGF-I receptor recruits a kinase termed CTK that phosphorylates SHPS-1 leading to formation of a multicomponent signaling complex which results in AKT and MAP kinase activation [16]. In the case of vascular smooth muscle cells, this results in dedifferentiation and growth stimulation [17], and in the case of endothelial cells, it results in dysfunctional cell behavior, abnormal cell-cell contacts, reduction in cell junction proteins, and an increase capillary leakage [18].

Our laboratory has shown that SHPS-1 is not phosphorylated in response to IGF-I unless it binds to a cellular membrane protein termed integrin-associated protein (IAP) or CD-47 [19]. This does not occur in vascular cells under normoglycemic conditions because IAP is cleaved constitutively. However, following exposure to hyperglycemia, cleavage is inhibited [20]. Since the SHPS-1 binding site on IAP is contained within the cleaved fragment, inhibition of cleavage results in a major increase in IAP/SHPS-1 association thereby facilitating IGF-I stimulation of pathophysiologic changes. Based on those studies, we utilized a monoclonal antibody (B6H12) to disrupt IAP/SHPS-1 and showed that it attenuated IGF-I signaling and prevented an increase in retinal vascular permeability in diabetic rats [18]. Therefore, this study was undertaken to determine whether a specific antibody prepared against a known IAP binding site for SHPS-1 could reproduce the effects of the monoclonal antibody that was not specifically directed against the SHPS-1 binding site and whether administration of this antibody after diabetic retinopathy had been established could reverse the early changes that occur in capillary permeability. Furthermore, we wished to determine whether the anti-IAP antibody was active following intraocular injections and whether it could attenuate the pathophysiologic changes that occur in IGF-I signal transduction in vivo.

## 2. Materials and Methods

Human IGF-I was a gift from Genentech (South San Francisco, CA). Streptomycin and penicillin were purchased from Life Technologies (Grand Island, NY). Antibodies against phospho-AKT (S473), AKT, phospho-Erk1/2, and Erk1/2 were purchased from Cell Signaling Technology Inc. (Beverly, MA). Antibodies against pY99 and VWF were purchased from Santa Cruz Biotechnology, Inc. (Santa Cruz, CA). An anti-VEGF antibody was purchased from Thermo Fisher Scientific (Waltham, MA). An anti-SHPS-1 antiserum was prepared in-house as described previously [21]. The monoclonal anti-IAP antibody, B6H12, was prepared as described previously [19]. The horseradish peroxidase-conjugated mouse anti-rabbit, goat anti-mouse, and mouse anti-rabbit light chain-specific antibodies were purchased from Jackson ImmunoResearch Laboratories (West Grove, PA). All other reagents were obtained from Sigma unless otherwise stated.

### 2.1. IAP Antibody Preparation

An anti-IAP antiserum was raised by our lab in a rabbit using the sequence CGNKNSTTREQN linked to keyhole limpet hemocyanin as an immunogen. Purified rabbit IgG was obtained via a protein A affinity column. An F(ab)2 fragment of the antibody was prepared using a F(ab)2 Preparation Kit following the manufacturer's instructions (Thermo Fisher Scientific, Cat 44988).

### 2.2. Cell Culture

Primary rat endothelial cells (RECs) were obtained from Cell Applications (San Diego, CA). The cells were cultured in rat endothelial cell growth medium (Cat# R211-500, Cell Applications) containing 25 mM glucose (Cat# G8769, Sigma) until confluency then incubated with rat endothelial cell basal medium (Cat# R210-500, Cell Applications) with 25 mM glucose overnight. The next day, basal medium was applied and the indicated anti-IAP antibody concentration was added for 2 h or an indicated time. IGF-I (100 ng/ml) was added 10 min before the cells were harvested.

### 2.3. Cell Proliferation Assay

RECs were plated on a 24-well plate with cell growth medium containing 25 mM glucose. After 24 h, serum-free basal medium with or without IGF-I (50 ng/ml) or anti IAP antibody (5-20 ug/ml) was added. After another 48 h, cells were counted.

### 2.4. Immunoprecipitation and Immunoblotting

The cell monolayers were lysed in a modified radioimmunoprecipitation assay (RIPA) buffer. Total cellular protein in the lysates was determined using BCA (Thermo Fisher Scientific, Rockford, IL). Immunoprecipitation was performed by incubating 0.5 mg of cell/retinal lysate protein with 1 *μ*g of anti-SHPS-1 or anti-pY99 antibody at 4°C overnight. Immunoblotting was performed using a dilution of 1 : 1000 for anti-pAKT (Ser473), pErk1/2, Erk1/2, and Shc antibodies; a dilution of 1 : 5000 for anti-SHPS-1 and *β*-actin antibodies; a dilution of 1 : 500 for anti-pY99 and IAP antibodies; and a dilution of 1 : 200 for anti-VWF antibody. The proteins were visualized using enhanced chemiluminescence (Thermo Fisher Scientific, Rockford, IL). All gel electrophoresis experimental results were analyzed using scanning densitometry. The scanning units obtained from the band of interest/loading control from three separate experiments were used for data analysis form the *in vitro* studies. For the in vivo studies, the results of each individual animal sample were analyzed in the same manner.

### 2.5. Diabetic Rat Study Design

All of the animal studies were reviewed and approved by the Institutional Animal Care and Use Committee of the University of North Carolina at Chapel Hill. Male Sprague-Dawley rats (Charles River, Wilmington, MA, USA) were housed under 12–12 h light-dark conditions, with free access to food and water. Control rats received an injection of vehicle. Hyperglycemia was induced by injection of streptozotocin via intraperitoneal (i.p.) injection (50 mg/kg body weight). Hyperglycemia (serum glucose > 250 mg/dl) was confirmed 6 days later. Glucose concentrations were maintained between 250-499 mg/dl using insulin via i.p. injection (9-15 U/kg daily; NPH insulin, Novo Nordisk, Princeton, NJ, USA). The glucose concentrations and body weight changes during the studies were included (Supplemental Figure 1A–1D). For the diabetic retinopathy reversal study, diabetic rats (*N* = 16) were allowed to remain hyperglycemic for 4 weeks before receiving an intraperitoneal injection of control F(ab)2 (*N* = 8) or anti-rat IAP F(ab)2 (*N* = 8) every 72 h. The injections were continued for 6 additional weeks. Four nondiabetic rats served as normal controls. During the prevention study, the rats were remained in hyperglycemic state for 9 days, then 11 diabetic rats were injected intraocularly with control F(ab)2 (35 *μ*g), and an additional 9 diabetic animals were injected with anti-rat IAP F(ab)2 (35 *μ*g). The animals were maintained for another 3 weeks and remained hyperglycemic during that time before being sacrificed. Nine nondiabetic rats served as normal controls. To obtain retinal extracts for analysis of biochemical changes, three groups of rats: nondiabetic (*N* = 5), diabetic receiving control antibody (*N* = 7), and diabetic receiving ant-IAP antibody (*N* = 8), were prepared following the same diabetic retinopathy preventive study protocol. After the animals were sacrificed, the retinas were isolated and extracts were prepared with modified radioimmunoprecipitation assay (RIPA) buffer in the presence of protease inhibitors (10 *μ*g/ml aprotinin, 1 *μ*g/ml leupeptin, 1 mM phenylmethylsulfonyl fluoride, and 1 *μ*g/ml pepstatin) and phosphatase inhibitors (25 mM sodium fluoride and 2 mM sodium orthovanadate). After sonication, the extracts were centrifuged at 14,000×*g* for 10 min at 4°C. The protein concentration of each extract was determined using a BCA assay (Thermo Fisher Scientific, Rockford, IL, USA). The activation of downstream signaling components was analyzed following the procedures described in immunoprecipitation and immunoblotting sections. Due to technical problems, 8 of 10 eyes in normal rats, 10 of 14 eyes in diabetic rats receiving control antibody, and 12 of 16 eyes receiving the anti-IAP antibody were of sufficient quality that they could be used for detection.

### 2.6. In Vivo Measurement of Vascular Permeability

Rats were anesthetized with ketamine (60 mg/kg body weight)/xylazine (9 mg/kg) cocktail. Once deep anesthesia had been achieved, warmed Evans blue (45 mg/kg; Fisher Scientific, Pittsburgh, PA, USA) was injected into the tail vein. Evans blue dye binds to albumin, allowing the measurement of albumin leakage from the vasculature. After 2 h, a lethal dose of ketamine (100 mg/kg) was administered. Blood was collected and centrifuged at 12,000×*g* for 5 min. The rats were perfused with 1% (wt/vol.) paraformaldehyde in citrate, and then, the eyes were removed and placed in PBS. The retinas were removed under a dissecting microscope and lyophilized, then resuspended in formamide and incubated at 70°C. After 18 h, the retinas in formamide were centrifuged at 13,000×*g* for 10 min before measuring Evans blue concentration.

A standard curve was generated using serial dilutions of Evans blue. The concentration of Evans blue in plasma or retinal extracts (2 *μ*l of each) was measured using a NanoDrop spectrophotometer (Thermo Fisher Scientific, Rockford, IL, USA) with excitation and emission wavelengths of 620 and 740 nm, respectively. The amount of Evans blue permeation from each retina was calculated as follows: Evans blue in the retina (*μ*g)/Evans blue in plasma (*μ*g/*μ*l)/retina dry weight (gm)/circulation time, and expressed as %/g/hr.

### 2.7. Optical Coherence Tomography (OCT) Measurement

The retinal thickness of all rats, including nondiabetic and diabetic rats with different treatments, was determined via OCT measurement using Micron IV (Phoenix Research Lab, Pleasanton, CA). Briefly, rats were anesthetized and pupils were dilated with 1% tropicamide. Corneas were moistened with GenTeal lubricant eye gel (Novartis) and positioned with the Micron eyepiece in direct contact with the eye through the gel. OCT images were captured using the full-scan setting as an average of 10 frames/scan. Three images were taken per eye in the superior/inferior and nasal/temporal directions relative to the optic nerve [22]. For the diabetic retinopathy reversal study, rats were scanned basally and after 4 and 10 weeks (at the end of the study). For the diabetic retinopathy prevention study, the rats were scanned at the time of antibody injection and 3 weeks later at the end of the study. The thickness of the retina was measured using ImageJ (v.1.51K, NIH) by measuring 10 sites of retinal thickness in a double-blinded manner.

### 2.8. Intraocular Injection

Each animal was anesthetized using ketamine/xylazine (60 mg/kg/9 mg/kg), then placed under a dissecting microscope. A 30 G needle was used to scrape away the conjunctiva in order to expose a bare sclera. The tip of a 30 G needle was inserted into the eye approximately 0.5 mm posterior to the limbus. The 30 G needle was removed, and a 33 G intraocular needle (NanoFil, World Precision Instruments, Sarasota, FL) was inserted into the vitreous at a 45° angle and the antibodies injected in a volume of 5 *μ*l.

### 2.9. Statistical Analysis

Densitometry results are expressed as the mean ± standard deviation (SD). All experiments were replicated at least three times to assure reproducibility. All experiment results were included to analyze for statistically significant differences using Student's *t*-test (in vitro experiments) or analysis of variance (ANOVA) followed by Tukey's post hoc multiple comparison test (in vivo experiments). Statistical significance was set at *p* < 0.05.

## 3. Results

**Figure 1:**
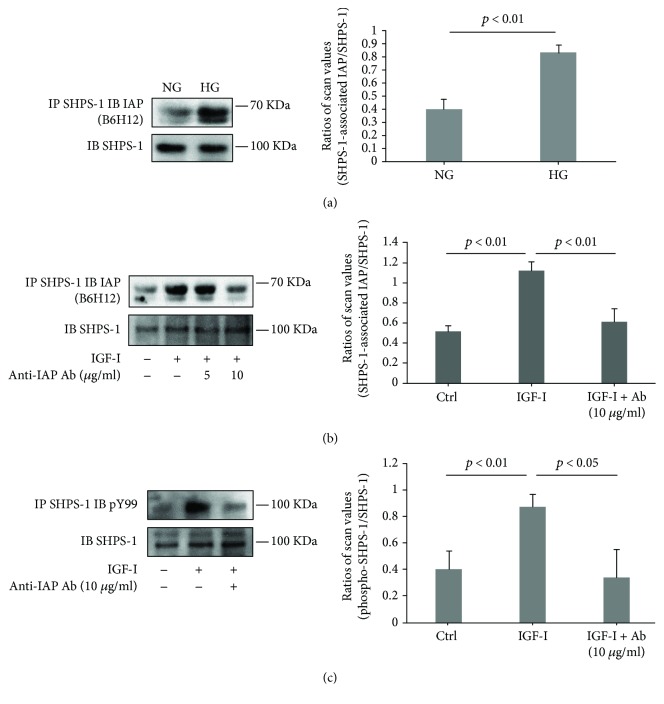
Anti-IAP antibody prevents IGF-I-stimulated SHPS-1/IAP association and SHPS-1 tyrosine phosphorylation. (a) Rat endothelial cells were grown to confluency in normal glucose (NG, 5 mM) or high glucose (HG, 25 mM) medium. Cells were exposed to serum containing growth medium before being harvested. (b, c) Cells were grown to confluency in HG medium, then placed in serum-free HG medium. The indicated concentrations of anti-IAP antibody were incubated with the cells for 2 h, and IGF-I (100 ng/ml) was incubated for 10 min before the cells were harvested. Cell lysates were immunoprecipitated with an anti-SHPS-1 antibody and immunoblotted with an anti-IAP antibody (a, b) or an anti-pY99 antibody (c). The same amount of cell lysate was immunoblotted with an anti-SHPS-1 antibody as a loading control. Each experiment was repeated 3 times, and the results were similar to the representative immunoblots shown in the figures. The bar graph shows the ratios ± SD of scanning densitometry values of SHPS-1-associated IAP (a, b) or phospho-SHPS-1 (c) divided by total SHPS-1. *p* < 0.05 and *p* < 0.01 indicate the significant differences.

**Figure 2:**
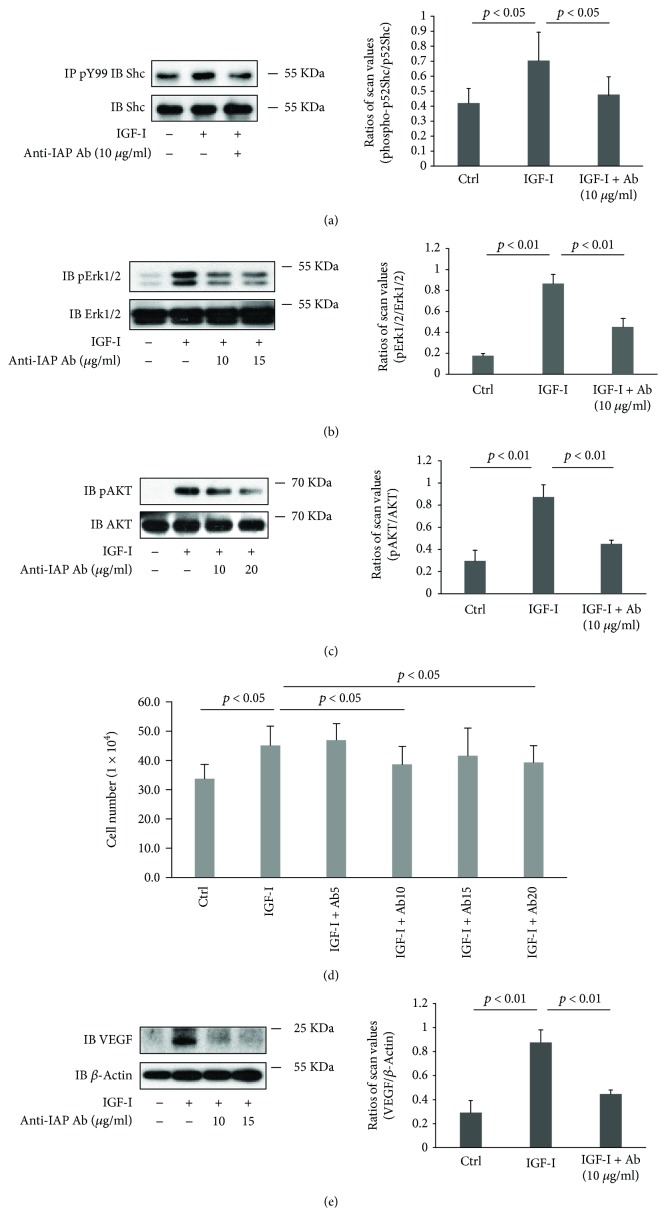
Disruption of IAP/SHPS-1 association attenuated IGF-I downstream signaling and IGF-I-stimulated cell proliferation. Rat endothelial cells were grown to confluency in high-glucose (HG, 25 mM) medium then placed in serum-free HG medium. The indicated concentration of anti-IAP antibody was incubated with the cells for 2 h, and IGF-I (100 ng/ml) was added for 10 min before the cells were harvested. (a) Cell lysates were immunoprecipitated with an anti-pY99 antibody and immunoblotted with an anti-Shc antibody. The same amount of cell lysate was immunoblotted with an anti-Shc antibody. The bar graph shows the ratios ± SD of scanning densitometry values of phospho-Shc divided by total Shc. *p* < 0.05 indicates the significant differences. The cell lysates were also immunoblotted with anti-pErk1/2 or Erk1/2 antibody (b) and anti-pAKT or AKT antibody (c). The bar graph shows the ratios ± SD of scanning densitometry values of pErk1/2 divided by total Erk1/2 (b) or pAkt divided by total Akt (c). *p* < 0.01 and *p* < 0.001 indicate the significant differences. (d) Cell proliferation assays were performed following a procedure described in Materials and Methods. *p* < 0.05 indicates the significant difference between two treatments. The results are the mean ± SD of three separate experiments with 3 determinations of each data point in each experiment. (e) Cell lysate was immunoblotted with anti-VEGF or *β*-actin antibody. The bar graph shows the ratios ± SD of scanning densitometry values of VEGF divided by *β*-actin. *p* < 0.01 indicates a significant difference. Each experiment was repeated 3 times, and the results were similar to the representative immunoblots shown in the figures.

To determine if the antibody directed against rat IAP would inhibit pathophysiologic changes that are stimulated by IGF-I and high glucose concentrations, we conducted several experiments in cultured primary RECs. Initially, we determined if the F(ab)2 fragment of the anti-IAP antibody would disrupt IAP/SHPS-1 association. In the presence of high glucose, there was a significant increase in the amount of IAP bound to SHPS-1 as compared to normal glucose (e.g., 2.2 ± 0.3-fold increase, *p* < 0.01, *N* = 3) (Figure 1(a)) and the anti-IAP antibody (10^−9^ M) inhibited IAP/SHPS-1 association (e.g., 87.9 ± 26.3% inhibition, *p* < 0.01, *N* = 3) (Figure 1(b)). In order to determine if this altered IGF-I signaling, we measured SHPS-1 phosphorylation. Exposure of RECs to high glucose and IGF-I resulted in an increased SHPS-1 phosphorylation (e.g., 2.2 ± 0.6-fold, *p* < 0.01), and the anti-IAP antibody inhibited this response by 60.9 ± 16.1% (*p* < 0.05) (Figure 1(c)). Based on this result, we determined if the anti-IAP antibody would alter downstream signaling. The aberrant increase in SHPS-1 phosphorylation that occurs in response to hyperglycemia in VSMC leads to both AKT and MAP kinase activation. Since MAP kinase phosphorylation increases in response to p52shc phosphorylation, we measured the ability of the anti-IAP antibody to inhibit IGF-I-stimulated p52shc tyrosine phosphorylation. As shown in Figure 2(a), the anti-IAP antibody (10 *μ*g/ml) inhibited this change significantly (e.g., 80.8 ± 14.0% inhibition, *p* < 0.05), and this inhibition was associated with a 60.1 ± 8.4% inhibition of ERK1/2 activation (*p* < 0.01) (Figure 2(b)). Similarly, phosphorylation of AKT in response to IGF-I was inhibited (e.g., 57.2 ± 11.9%, *p* < 0.05) (Figure 2(c)). Based on these findings, we determined if the anti-IAP antibody had an effect on cellular proliferation. As shown in Figure 2(d), in the presence of high glucose, IGF-I stimulated a 45% increase in proliferation and this was inhibited significantly by 20 *μ*g/ml of anti-IAP antibody.

Our prior studies have shown that IGF-I induces VEGF and VEGFR2 activation in cultured endothelial cells [18], and Smith et al. demonstrated that the effects of IGF-I on the retinal vasculature were mediated in part by VEGF induction [9]. Therefore, we determined if the anti-IAP antibody could inhibit VEGF secretion by RECs. The addition of IGF-I caused a 3.1 ± 0.9-fold (*p* < 0.01) increase in VEGF, and the anti-IAP antibody reduced this increase by 73.6 ± 16.0%, to a value that was comparable to control cells (*p*, NS) (Figure 2(e)).

**Figure 3:**
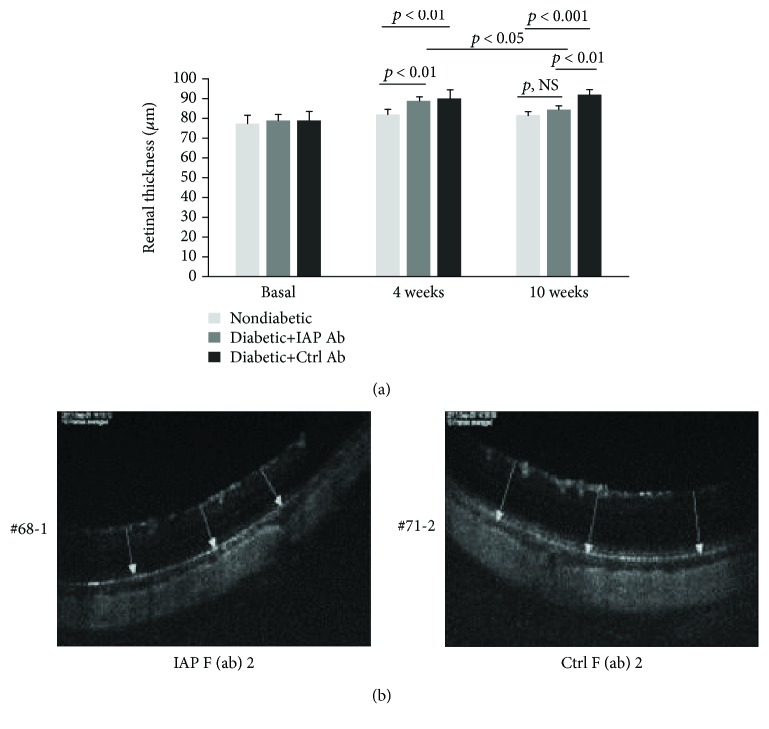
Disruption of IAP/SHPS-1 association reversed the hyperglycemia-induced increase in retinal thickness. (a) Diabetic animals were prepared and maintained for 4 weeks before they were injected intraperitoneally with the control or anti-IAP antibody for 6 weeks following the protocol for the diabetic retinopathy reversal study as described in Materials and Methods. Retinal thickness was measured basally, at 4 weeks (*N* = 4 for nondiabetic rats, *N* = 8 for diabetic rats with IAP antibody, and *N* = 8 for diabetic rats with control antibody) and at 10 weeks (*N* = 4 for nondiabetic rats, *N* = 5 for diabetic rats with IAP antibody, and *N* = 5 for diabetic rats with control antibody) following the procedure described in Materials and Methods. (b) Representative OCT images are shown from the control (Ctrl) F(ab)2- and IAP F(ab)2-treated animals. *p* < 0.05, *p* < 0.01, and *p* < 0.001 indicate significant differences between two treatments. *p*, NS indicates no significant difference between two treatments.

**Figure 4:**
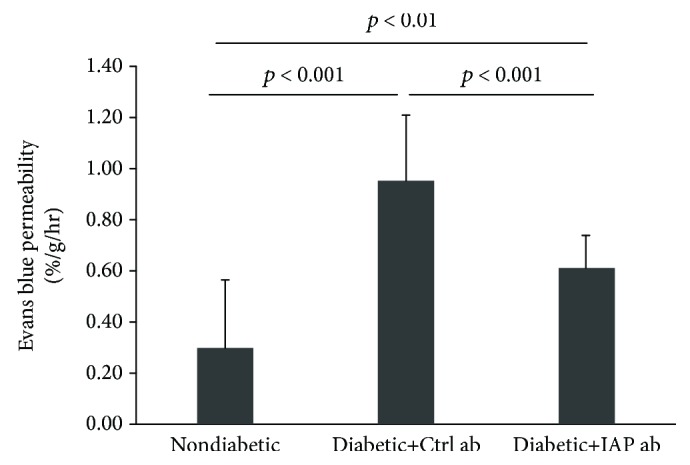
Disruption of IAP/SHPS-1 association prevented the hyperglycemia-induced increase in vascular permeability. The animals were treated following the protocol for diabetic retinopathy reversal study described in Materials and Methods. Evans blue permeation from the retinal vasculature was measured at 10 weeks (after 6 weeks of antibody exposure) (*N* = 3 for nondiabetic rats, *N* = 6 for diabetic rats with control antibody, and *N* = 6 for diabetic rats with IAP antibody) following the procedure described in Materials and Methods. *p* < 0.01 and *p* < 0.001 indicate significant differences between two treatments.

Initially, to determine if the anti-IAP antibody was active in vivo and if early changes of diabetic retinopathy such as increased capillary permeability that were established could be reversed following anti-IAP antibody treatment, rats were made diabetic with streptozotocin. After four weeks, optical coherence tomography was utilized to determine if there was a change in retinal thickness which is an indirect measure of increased capillary permeability. Retinal thickness was similar in the nondiabetic and diabetic groups at baseline. After four weeks, retinal thickness increased significantly to 89.9 ± 5.0 *μ*m in the diabetic rats compared to 82.3 ± 2.7 *μ*m in the nondiabetic rats (*p* < 0.01) (Figure 3). This value was similar to the value in the diabetic rats at baseline. Beginning at this time point, either control or active anti-IAP antibody was administered by intraperitoneal injection to the diabetic animals for 6 weeks. Retinal thickness continued to increase to 92.3 ± 2.5 *μ*m in the diabetic animals exposed to the control antibody, but the change was not significant when compared to the 4-week value (*p* = 0.28) (Figure 3). In contrast, retinal thickness decreased significantly to 84.6 ± 2.0 *μ*m in the animals treated with the active anti-IAP antibody (*p* < 0.01 compared to animals receiving control antibody and *p* < 0.05 compared to the same group of animals after 4 weeks of diabetes). This value was comparable to the nondiabetic animals who had a value of 82.2 ± 1.6 *μ*m (*p*, NS). To confirm that these changes were associated with a change in retinal vascular permeability, this parameter was measured in animals that received the control and active anti-IAP antibody after the six-week treatment. Animals that received the control antibody had a significantly higher retinal leakage as compared to the animals that received the active anti-IAP antibody (0.96 ± 0.25%/g/h versus 0.62 ± 0.12%/g/h, *p* < 0.001) (Figure 4). Although the retinal leakage was significantly lower in the animals treated with the active anti-IAP antibody, it was still greater than nondiabetic animals that were maintained for the same 10-week interval (0.62 ± 0.12%/g/h versus 0.30 ± 0.27%/g/h, *p* < 0.01). Therefore, the anti-IAP antibody partially reversed the change that occurred in capillary leakage but did not restore it to normal.

**Figure 5:**
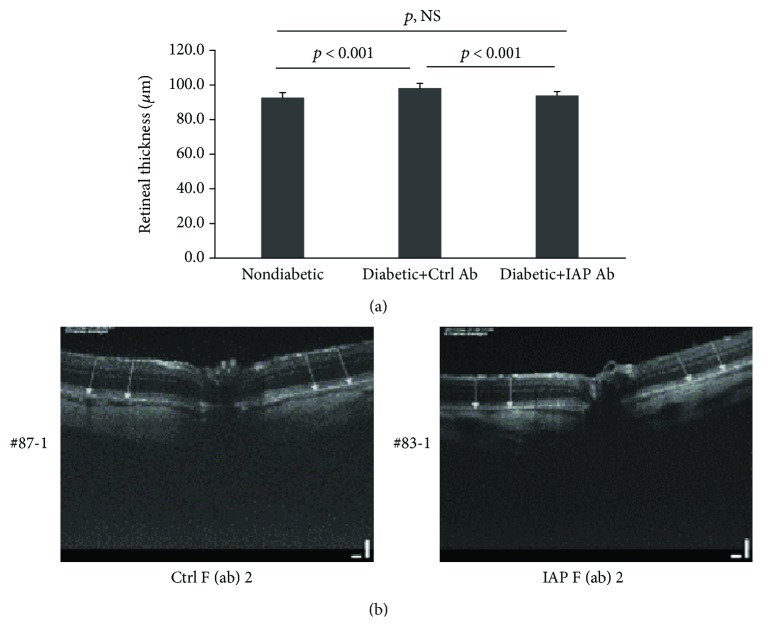
Disruption of IAP/SHPS-1 association via intraocular injection of an anti-IAP antibody prevented the hyperglycemia-induced increase in retinal thickness. (a) The animals were treated, and retinal thickness was measured after 3 weeks of treatment (*N* = 9 for each group) following the protocol for diabetic retinopathy preventive study described in Materials and Methods. (b) The representative OCT images are shown from the control (Ctrl) F(ab)2- and IAP F(ab)2-treated animals. *p* < 0.01 and *p* < 0.001 indicate the significant differences between two treatments. *p*, NS indicates no significant difference between the two treatments.

**Figure 6:**
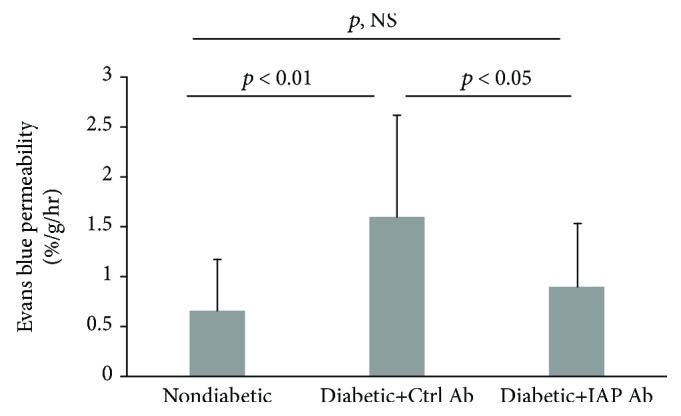
Disruption of IAP/SHPS-1 association via intraocular injection of an anti-IAP antibody prevented the hyperglycemia-induced increase in vascular permeability. The animals were treated following the protocol for the diabetic retinopathy preventive study described in Materials and Methods. Evans blue permeation from the retinal vasculature was measured after 3 weeks of treatment (*N* = 7 for nondiabetic rats and *N* = 9 for each group of diabetic rats with control antibody or anti-IAP antibody) following the procedure described in Materials and Methods. *p* < 0.01 indicates the significant difference between two treatments. *p*, NS indicates no significant difference between two treatments.

To determine whether the anti-IAP antibody was active following intraocular injection, additional rats were made diabetic. Following 8 to 10 days of hyperglycemia, the animals received intraocular injections (35 *μ*g per eye) of active or control antibody. Additional normal animals received a saline injection. A single time point analysis was conducted three weeks after the injection. The results showed that retinal thickness was increased, 6.4 ± 2.8% (*p* < 0.001), from baseline in the diabetic rats that received control antibody and that this increase was substantially greater than in the nondiabetic rats, −0.4 ± 2.6% (*p* < 0.001) (Figure 5). Treatment with the anti-IAP antibody prevented the increase in the diabetic rats (0.8 ± 2.5%; *p*, NS compared to nondiabetic rats). Analysis of Evans blue dye permeability showed that it increased from 0.67 ± 0.51%/g/h in the nondiabetic animals to 1.63 ± 0.99%/g/h in the diabetic animals receiving control antibody (*p* < 0.01). The active anti-IAP antibody inhibited this increase to 0.92 ± 0.62%/g/h (*p* < 0.001 compared to control antibody treatment and *p*, NS compared to nondiabetic rats) (Figure 6).

**Figure 7:**
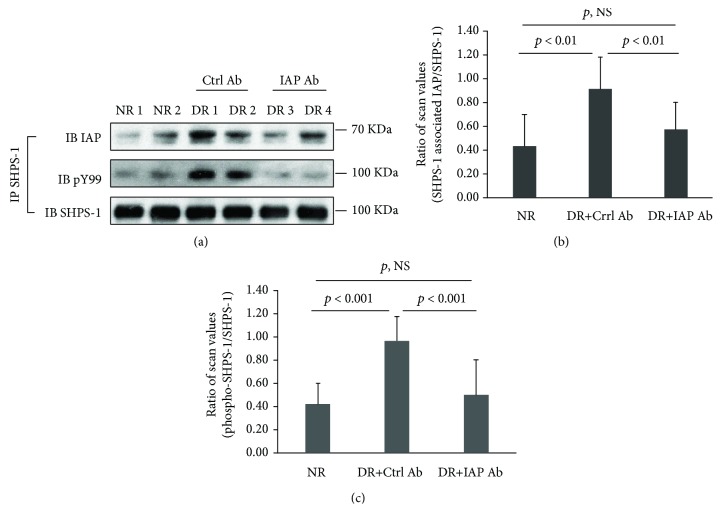
Intraocular injection of an anti-IAP antibody disrupted IAP/SHPS-1 association and attenuated IGF-I-stimulated SHPS-1 tyrosine phosphorylation in the retinas from diabetic rats. Animals (*N* = 4 for nondiabetic rats, *N* = 5 for diabetic rats with control antibody, and *N* = 6 for diabetic rats with IAP antibody) were treated, and retinal extracts were prepared following the protocol described in Materials and Methods. (a) The retinal extracts were immunoprecipitated with an anti-SHPS-1 antibody and immunoblotted with an anti-IAP or pY99 antibody. The blots were reprobed with an anti-SHPS-1 antibody as a loading control. The bar graphs show the ratio of scan values of SHPS-1-associated IAP divided by SHPS-1 (b) or pY99 divided by SHPS-1 (c). *p* < 0.01 and *p* < 0.001 indicate a significant difference between two treatments. *p*, NS indicates no significant difference. NR = normal rat; DR = diabetic rat.

**Figure 8:**
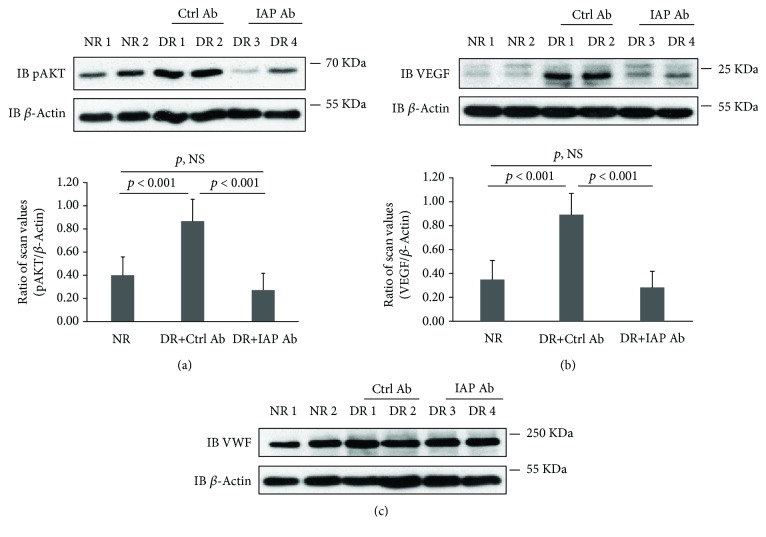
Disruption of IAP/SHPS-1 association via intraocular injection of an anti-IAP antibody attenuated IGF-I-stimulated AKT activation and VEGF expression in the retinas from diabetic rats. Animals (*N* = 4 for nondiabetic rats, *N* = 5 for diabetic rats with control antibody, and *N* = 6 for diabetic rats with IAP antibody) were treated, and retinal extracts were prepared following the protocol described in Materials and Methods. The retinal extracts were immunoblotted with an anti-pAKT (a) or VEGF (b) antibody. The blots were reprobed with anti-*β*-actin antibody as a loading control. The bar graphs show the ratio of scan values of pAKT band or VEGF band divided by *β*-actin band. *p* < 0.001 indicates the significant difference between two treatments. *p*, NS indicates no significant difference. (c) The retinal extracts were immunoblotted with an anti-VWF antibody. The blots were reprobed with anti-*β*-actin antibody as a loading control.

To determine if intraocular injection resulted in inhibition of aberrant IGF-I signaling, several biochemical parameters were measured in retinal extracts obtained at the time of sacrifice. Consistent with the in vitro data, IAP/SHPS-1 association was increased to 2.1 ± 0.3-fold (*p* < 0.01) in diabetic rats who received control antibody (Figure 7(a) and Supplemental Figure 2A–2D) [23]. Importantly, the anti-IAP antibody inhibited this association significantly (79.8 ± 22.3%, *p* < 0.01), to a level that was similar to nondiabetic rats (Figure 7(b)). Disruption of IAP/SHPS-1 association resulted in a significant reduction in SHPS-1 phosphorylation (e.g., 80.5 ± 23.4% reduction, *p* < 0.001) (Figure 7(c)). However, two eyes that had been injected with anti-IAP antibody had minimal inhibition of SHPS-1 phosphorylation (Supplemental Figure 2D) [23]. To determine if the active anti-IAP antibody had been degraded in these eyes, we immunoblotted vitreous for rabbit IgG. The findings confirmed that the levels of rabbit IgG were reduced in these eyes whereas those that had significant inhibition of SHPS-1 phosphorylation had much higher intact antibody levels (Supplemental Figure 3) [23]. Therefore, it is probable that in these eyes, the anti-IAP antibody concentration had been reduced to a level that was no longer active at the end of three weeks. Importantly, across the entire group of animals, the changes in SHPS-1 tyrosine phosphorylation paralleled the ability of the anti-IAP antibody to inhibit IAP/SHPS-1 association (Figure 7(a) and Supplemental Figure 2A–2D). Additionally, we measured downstream signaling parameters. As shown in Figure 8(a), AKT activation was significantly attenuated in the animals that had major inhibition of SHPS-1 phosphorylation (e.g., 74.3 ± 10.1% reduction, *p* < 0.01). Retinal extracts from the diabetic animals showed a major increase in VEGF compared to those for normal rats, and the animals that showed significant disruption of IAP/SHPS-1 association had a significant reduction in VEGF induction compared to control antibody-injected animals (Figure 8(b)) (e.g., 66.9 ± 18.8% reduction, *p* < 0.001). To confirm that the extracts contained endothelium, we immunoblotted the retinal extracts using an anti-von Willebrand factor antibody since this is an endothelial-specific protein. As shown in Figure 8(c), abundant VWF was present in the retinal extracts.

## 4. Discussion

The findings in this study show that disruption of IAP/SHPS-1 association results in prevention and reversal of the early changes that occur in diabetic retinopathy. Specifically, the rats exhibited typical early changes of diabetic retinopathy including retinal thickening, increased capillary leakage, and expression of proteins such as VEGF in the retina that are associated with diabetic retinopathy progression. Since IAP/SHPS-1 association is required to activate aberrant IGF-I-mediated signaling in retinal endothelial cells exposed to hyperglycemia, we reasoned that attenuation of this association would result in inhibition of these pathophysiologic changes. Importantly, these findings extend previous observations by showing that in rats with established changes in capillary thickness (an early indicator of retinal edema), there was significant reversal of retinal thickening. Although capillary leakage was not measured at 4 weeks, when it was quantified at 10 weeks, it was substantially lower compared to the animals that received control antibody, a finding consistent with either stabilization or reversal of the changes that had occurred by week 4.

A second major extension of previous findings is that the anti-IAP antibody is active following intraocular injection. Specifically, a single intraocular administration of the anti-IAP antibody resulted in drug levels that were adequate to maintain IAP/SHPS-1 disruption after three weeks in most of the animals. That this resulted in a relevant biochemical signaling change was demonstrated by showing that SHPS-1 phosphorylation was inhibited. Interestingly, the increase in SHPS-1 phosphorylation was sustained in the diabetic animals in spite of the fact that they were not administered IGF-I or any other growth factor known to stimulate this process. This suggests that ongoing hyperglycemia and ambient retinal IGF-I concentrations are sufficient to result in persistent activation of this pathway.

We have definitively demonstrated in VSMC that downregulation of IRS-1 leads to recruitment of a kinase (CTK) to the IGF-I receptor [16]. Following IGF-I receptor activation, CTK binds to a site on the IGF-I receptor that is identical to the site that binds IRS-1. Therefore, the reduction in IRS-1 results in enhanced recruitment of this kinase to the plasma membrane and once localized therein, CTK phosphorylates SHPS-1. Our studies have shown that in normoglycemic animals, SHPS-1 is not constitutively activated and this only occurs in response to persistent elevation in glucose concentrations [15, 17]. In this study, we extend that finding to show that in cultured primary endothelial cells and in the diabetic retina, there is upregulation of SHPS-1 tyrosine phosphorylation. This suggests that the same mechanism that is operative in VSMC is occurring in retinal endothelial cells resulting in constitutive activation of the IAP/SHPS-1 signaling complex.

Our prior studies had shown that in both cultured VSMC and endothelial cells, stimulation of SHPS-1 phosphorylation leads to assembly of a signaling complex based on the recruitment of the protein tyrosine phosphatase SHP-2 to phosphorylated SHPS-1 [23]. Following SHP-2 recruitment, a complex consisting of c-Src, Nox4, and p52Shc is assembled. Shc then recruits both Grb-2 and the p85 subunit of PI-3 kinase leading to activation of both AKT and MAP kinase [15, 24]. The activation of these pathways leads to dysfunctional endothelial behavior including downregulation of junctional signaling proteins, such as occludin, as well as upregulation of VEGF synthesis and/VEGFR2 activation [18]. These changes lead to loss of normal gap junction formation and enhanced endothelial cell permeability. However, for this pathway to be activated requires not only IRS-1 downregulation but also the association of IAP with SHPS-1. This association requires intact IAP [18]. Our prior studies showed that in VSMC and endothelium, IAP is cleaved constitutively and that the domain that binds to SHPS-1 is removed following cleavage [18, 20]. In contrast, in the presence of hyperglycemia, this binding domain is retained and this allows SHPS-1 to be phosphorylated by CTK. Our findings in this study demonstrate not only that this process is activated in retina in vivo but that its activation persists for at least three weeks following the induction of hyperglycemia. Furthermore, we demonstrated that our antibody which disrupts IAP/SHPS-1 association could markedly inhibit SHPS-1 phosphorylation and that the degree of inhibition was proportionate to the degree of SHPS-1/IAP disruption. Therefore, we conclude that injection of this anti-IAP antibody holds promise for halting the progression of diabetic retinopathy even in the presence of persistent hyperglycemic stimulation of these pathophysiologic changes.

These results also extend prior studies since they show that the downstream signaling events that occur in response to IGF-I and hyperglycemia that have been characterized in endothelial cells in vitro are present in the diabetic retina. These signaling events such as AKT activation and induction of VEGF have been linked to pathophysiologic changes that occur in DR [25, 26]. Importantly, the anti-IAP antibody inhibited these changes providing further evidence that it is functioning to inhibit signaling events that have been linked to retinopathy progression.

The role of IGF-I in diabetic retinopathy has been studied using several different model systems. IGF-I protects human retinal endothelial cells from apoptosis and enhances proliferation in vitro [23, 27]. Notably, IGF-I is required for normal retinal vascular development, and humans with mutations in either the growth hormone receptor or the IGF-I gene have lower numbers of vascular branching points compared to reference controls [28]. Multiple studies in animal models support the conclusion that enhanced IGF-I production accelerates retinopathy and that increases in IGF-I within the retina are more important than systemic changes in plasma IGF-I concentrations for the development of proliferative DR [29–31]. Inhibition of retinal IGF-I synthesis following administration of somatostatin analogues to diabetic mice inhibited retinal neovascularization, and direct injection of IGF-I reversed this effect indicating that it was independent of growth hormone [32]. Furthermore, knockout of the insulin and IGF-I receptors in vascular endothelial cells in mice protected against hypoxia-induced retinal neovascularization [10]. These mice also maintained normal vascular permeability following injury [33]. Transfection of proliferating endothelial cells with a ribozyme that inhibited IGF-I receptor expression inhibited neovascularization in response to hypoxia or injury [34]. An additional study determined that following intravenous administration of IGF-I, the increase of IGF-I in vitreous resulted in increased retinal AKT, JNK, HIF1*α*, NF-kappa B, and VEGF expression, and an IGF-I receptor blocking antibody inhibited these changes [35]. Direct injection of high concentrations of IGF-I into pig vitreous induced capillary basement membrane thickening, microaneurysms, and capillary leakage [36]. The most definitive animal model demonstrating the effect of IGF-I is the transgenic mouse model. These animals overexpress IGF-I in the retina and develop multiple changes consistent with diabetic retinopathy including increased capillary leakage, retinal thickening, pericyte dropout, development of acellular capillaries, and proliferative retinopathy [11]. Importantly, the most aged animals with advanced disease showed retinal detachment. An additional study in these animals showed that pericyte dropout was associated with increased vascular permeability, altered intracellular adhesions, decreased vascular junction integrity, and increased VEGF and with a breakdown of the blood-brain barrier [36]. An equivalent increase in systemic IGF-I levels did not reproduce these effects indicating that the local concentration change in the retina was driving these responses.

Studies in humans have shown that IGF-I concentrations in vitreous samples removed from patients with proliferative retinopathy were 2.5-3-fold higher than those from nondiabetics [29, 30, 37] and there was a relationship between the degree of IGF-I elevation and the severity of proliferative changes. Increased IGF-I during the third trimester of pregnancy is associated with progression of diabetic retinopathy in women with type I diabetes, and subjects with higher IGF-I levels have a twofold greater likelihood of retinopathy progression independent of hemoglobin A1c [38]. Human studies have shown that polymorphisms containing multiple CA repeats that increase serum IGF-I levels are associated with 2.8-fold increase in risk of proliferative retinopathy in type 2 diabetics [39]. An additional study showed that following gastric bypass surgery in subjects with type II diabetes, the levels of bioactive IGF-I were increased to 50% in those subjects with proliferative retinopathy [40]. Multivariate linear regression showed that bioactive IGF-I was the parameter that correlated most closely with the presence of this complication.

IGF-I and VEGF function together to regulate retinal blood vessel development, and IGF-I knockout mice do not have normal vessel development even if normal VEGF levels are present [13]. Administration of IGF-I restores vessel growth, and both VEGF and IGF-I are required for optimal AKT activation in mouse endothelium in vivo. Multiple studies have shown that IGF-I increases VEGF expression in the retina. Punglia et al. demonstrated that IGF-I stimulated VEGF expression by Muller cells and this was dependent upon NF-kappa B and HIF-1*α* induction [12]. Additional studies have demonstrated that the VEGF expression is regulated by IGF-I, and blockade of the IGF-I receptor with an anti-receptor antibody inhibited VEGF increases. Hellstrom et al. demonstrated that injection of an IGF-I receptor antagonist into mouse models of retinal neovascularization resulted in an inhibition of the ability of IGF-I to induce VEGF expression as well as attenuation of induction of MAP kinase and retinal angiogenesis [13]. The connection between IGF-I and VEGF was further strengthened by a study which showed that microRNA 18b which is expressed in endothelial cells was downregulated by hyperglycemia. This resulted in upregulation of VEGF secretion and promoted retinal endothelial cell proliferation. The target of microRNA 18b was shown to be IGF-I, and IGF-I could antagonize the effect of this microRNA by increasing VEGF production and cell proliferation [41]. Whether VEGF mediates all of the effects induced by IGF-I was addressed by Deissler et al. who showed that blocking VEGF actions with an anti-VEGF antibody blocked IGF-I-simulated endothelial cell migration but not proliferation [42].

There are significant limitations to the interpretation of our studies. Specifically, the studies had a relatively short duration and longer-term experiments will be required to determine if these effects can be sustained over an extended time period following repetitive intraocular injections. Furthermore, pathophysiologic events that occur later in the course of diabetic retinopathy such as formation of acellular capillaries and pericyte dropout require studies of longer duration to document whether disruption of IAP/SHPS-1 can attenuate these processes. Finally, we did not measure neovascularization since this does not occur in the rat model of diabetic retinopathy. However, previously, we showed that injection of an anti-IAP antibody that disrupted IAP/SHPS-1 inhibited neovascularization in newborn rats using the retinopathy of prematurity model [18].

The role of IAP in mediating diabetic retinopathy has received minimal analysis. One study showed that epiretinal membranes isolated from patients with proliferative retinopathy and maintained in culture contained cells that when exposed to high glucose increased their expression IAP and VEGFR2 further suggesting that IAP may be involved in the pathogenesis of this condition [43]. Thrombospondin-2 (TSP-2) is a protein that binds directly to IAP and enhances IAP-mediated cellular responses [44]. Experimental rats with diabetes have increased expression of TSP-2 in the retina [45]. Importantly, thrombospondin-2 is highly expressed in humans with proliferative retinopathy and epiretinal membranes isolated from these patients showed a significantly higher number of blood vessels expressing IAP and TSP-2 in membranes with active neovascularization compared to those with quiescent disease [45].

In summary, diabetic retinopathy is a chronic condition associated with multiple pathophysiologic events. In this study, we have outlined a unique pathway by which IGF-I contributes to the pathogenesis of this condition. The key molecular event appears to be inhibition of cleavage of IAP resulting in signaling through an alternative pathway mediated through the transmembrane protein SHPS-1. The increased concentrations of IGF-I that are known to occur in the diabetic retina are sufficient to activate this pathway which results in increased VEGF expression and the induction of pathophysiologic events that are known to result from stimulation of the VEGFR2 receptor. Additionally, IGF-I can directly stimulate many of these events such as AKT and MAP kinase activation through its receptor. Therefore, targeting the IAP/SHPS-1 interaction has the potential to inhibit pathophysiologic events that occur in response to both of these growth factors during the development of this condition.

## Data Availability

The data used to support the findings of this study are available from the corresponding author upon request.

## Supplementary Materials

Supplementary Materials Supplemental Figure 1: body weight and serum glucose changes in the reversal and prevention studies. Supplemental Figure 2: intraocular injection of an anti-IAP antibody disrupted IAP/SHPS-1 association and attenuated IGF-I-stimulated SHPS-1 tyrosine phosphorylation in the retinas from diabetic rats. Supplemental Figure 3: a low level of remaining anti-IAP antibody was associated with less inhibition of SHPS-1 tyrosine phosphorylation.
