# Supplementary material for: Inhibition of Aberrant IGF-I Signaling in Diabetic Male Rat Retina Prevents and Reverses Changes of Diabetic Retinopathy
Source: J Diabetes Res. 2019 Mar 27;2019:6456032. doi: 10.1155/2019/6456032 (PMC6458945; doi:10.1155/2019/6456032)
Supplement: Supplementary Materials — Supplemental Figure 1: body weight and serum glucose changes in the reversal and prevention studies. Supplemental Figure 2: intraocular injection of an anti-IAP antibody disrupted IAP/SHPS-1 association and attenuated IGF-I-stimulated SHPS-1 tyrosine phosphorylation in the retinas from diabetic rats. Supplemental Figure 3: a low level of remaining anti-IAP antibody was associated with less inhibition of SHPS-1 tyrosine phosphorylation. [file 6456032.f1.pdf]

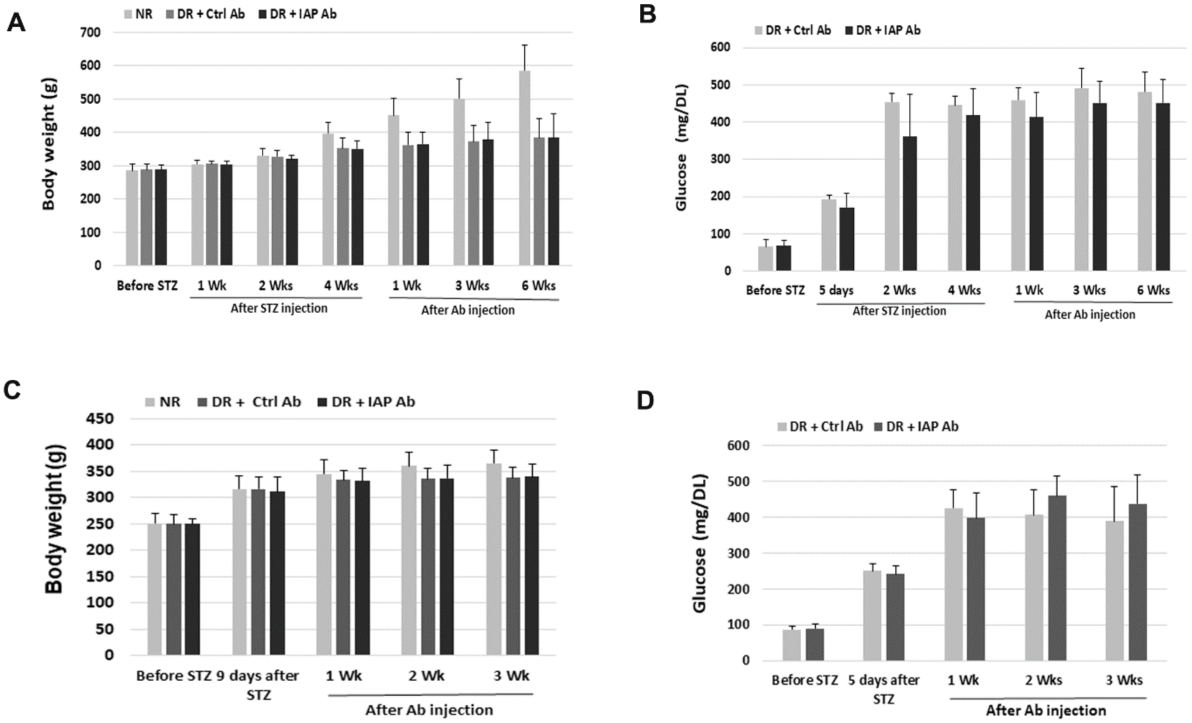

Supplemental Figure 1

Supplemental Figure 1. Body weight and serum glucose changes in the reversal and prevention studies. Body weights were obtained at the indicated time points for the reversal (A) or prevention studies (C). Serum glucose levels were obtained at the indicated time points for the reversal (B) or the preventive studies (D). The values were means of average ± SD.

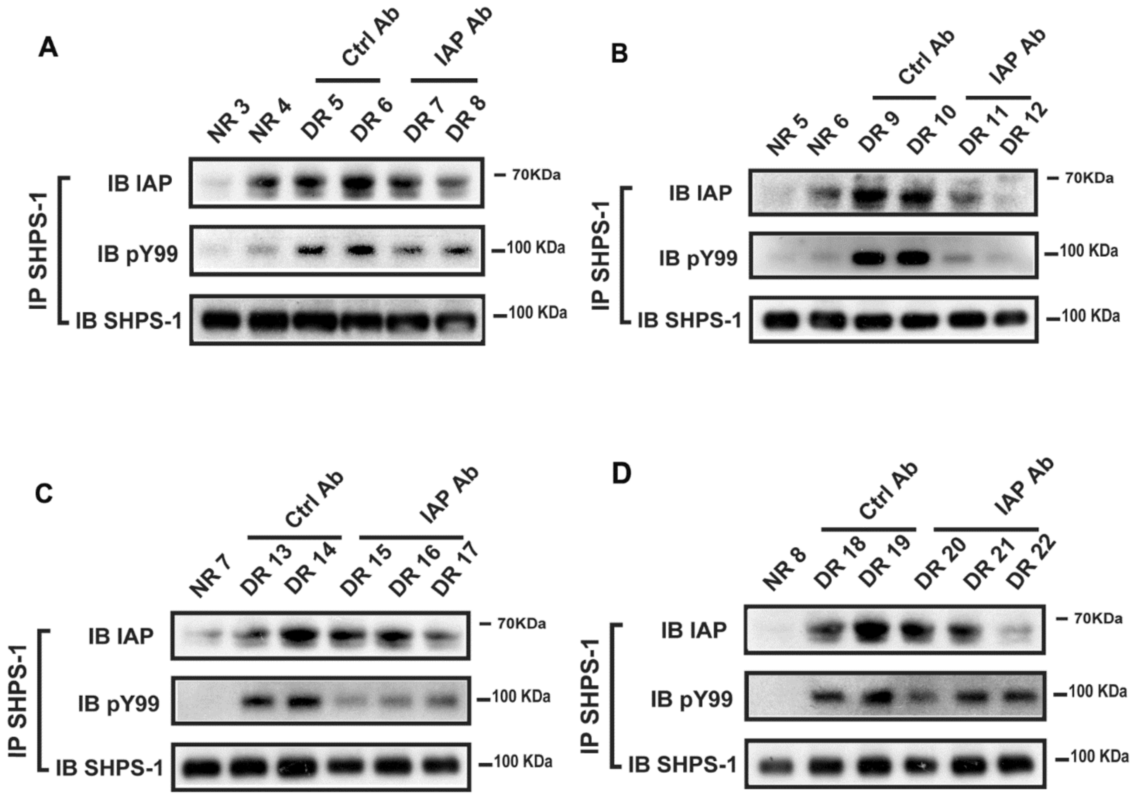

Supplemental Figure 2

Supplemental Figure 2. Intraocular injection of an anti-IAP antibody disrupted IAP/SHPS-1 association and attenuated IGF-I-stimulated SHPS-1 tyrosine phosphorylation in the retinas from diabetic rats. Animals were treated and retinal extracts were prepared following the protocol for biochemical analyses described in the Materials and Methods. (A, B, C, D) The retinal extracts were immuoprecipitated with an anti-SHPS-1 antibody and immunoblotted with an anti-IAP or pY99 antibody. The blots were reprobed with an anti-SHPS-1 antibody as a loading control.

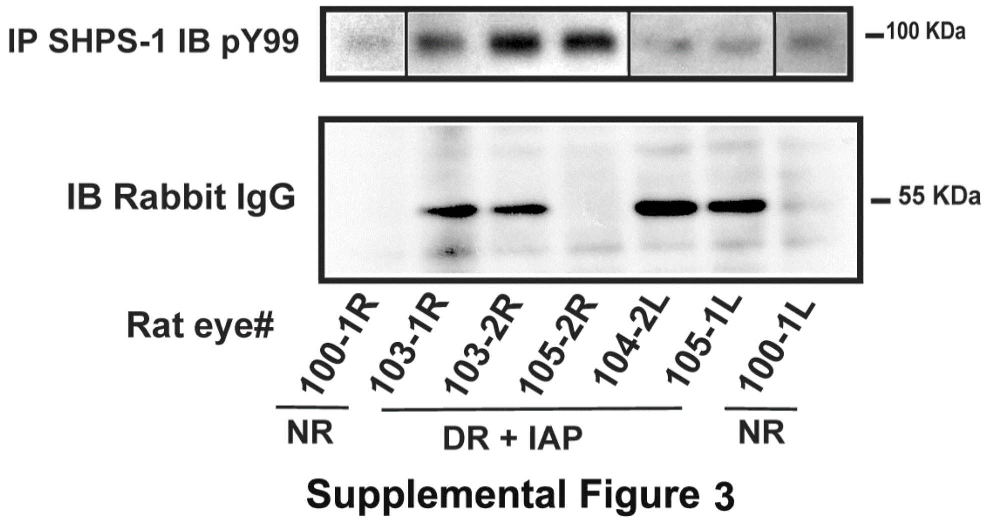

Supplemental Figure 3. Low remaining of anti-IAP antibody was associated with a less inhibition of SHPS-1 tyrosine phosphorylation. Animals were treated and retinal extracts were prepared following the protocol for retinal biochemistry study described in the Materials and Methods. The retinal extracts were immuoprecipitated with an anti-SHPS-1 antibody and immunoblotted with an anti-IAP or pY99 antibody. The vitreous obtained from the corresponding eyes were immunoblotted with an anti-rabbit IgG antibody.
